# Did Expanded Dental Insurance Improve Chewing Ability in the Older Korean Population? Results of an Interrupted Time-series Analysis

**DOI:** 10.2188/jea.JE20200417

**Published:** 2022-05-05

**Authors:** Nam-Hee Kim, Jarvis T. Chen, Ichiro Kawachi

**Affiliations:** 1 Department of Dental Hygiene, Yonsei University, Wonju College of Medicine, Wonju, Gangwon-do, Republic of Korea; 2 Department of Social and Behavioral Sciences, Harvard T.H. Chan School of Public Health, Boston, MA, USA

**Keywords:** dental insurance, chewing ability, interrupted time-series analysis, quasi-experimental, older adults, causal inference

## Abstract

**Background:**

In 2012, the Korean National Health Insurance extended its coverage to include denture services for older adults. We examined whether the new policy resulted in improved chewing ability in the eligible population.

**Methods:**

We used interrupted time-series (ITS) analysis, a quasi-experimental design, to analyze the effect of the policy. We used data from the Korea National Health and Nutrition Examination Survey conducted from 2007 to 2016–2018. The study population consisted of two groups: the treatment group, aged 65 years or older and eligible for the dental insurance benefit; and the control group, those younger than 65 years and ineligible. The main evaluated outcome was self-reported chewing difficulty.

**Results:**

The ITS analysis showed that chewing difficulty decreased annually by 0.93% (95% CI, −1.30 to −0.55%) and 0.38% (95% CI, −0.59 to −0.16%) after the policy extension in the older than 65 and younger than 65 groups, respectively. However, we could not conclude that the insurance extension affected chewing difficulty because there was a decrease in the control group as well.

**Conclusion:**

Chewing ability improved in both older and younger adults regardless of dental insurance coverage for older adults. Other exogenous factors probably led to the improvements in chewing ability as well as dental insurance benefits.

## INTRODUCTION

Worldwide, older adults suffer from chewing difficulty, which affects their quality of life and wellbeing. Globally, poor older people are more likely to have serious oral problems due to economic barriers to dental care, resulting in higher burdens of disease.^1^^–^^5^

South Korea launched a dental insurance expansion for older adults in 2012 under their national health care plan. Individuals aged 65 years and older became eligible for elderly dental insurance benefits, resulting in savings of approximately 50–70% for out-of-pocket expenses related to dentures and dental implants. Dental scaling has been covered for all adults since 2013, encouraging access to preventive dental care for those populations.

However, mixed results have been reported with regard to dental insurance benefits. For example, in the South Korean context, insurance expansion was shown to improve dental visits and self-reported oral health.^6^^–^^8^ In addition, expanded dental insurance for older adults was shown to alleviate the cost burden of treatment in this age group.^9^ On the other hand, significant out-of-pocket cost payments remain for low income older adults.^10^ Studies have also indicated inconsistent findings regarding the impact of insurance expansion on socioeconomic inequalities in oral health.^7^^,^^11^^,^^12^ Dental care access improved for higher socioeconomic groups after the insurance expansion,^11^^,^^12^ resulting in persistent oral health inequalities on the relative scale (even though inequalities on the absolute scale shrank).^7^

However, the findings of previous studies were not based on a strong causal identification strategy. For example, they did not attempt to address the endogeneity of extended dental insurance (ie, reverse causality or unmeasured confounding) by estimating the effects of coverage changes.

In the present study, we sought to implement a quasi-experimental approach by evaluating the effects of a policy change that was plausibly exogenous, or unrelated to health and to all observed or unobserved predictors of both coverage and health outcomes.^13^^,^^14^

Focusing on older adults, there have been two studies that implemented a quasi-experimental regression discontinuity design to examine the effect of insurance expansion on oral health outcomes. Despite cost sharing for dentures and an increased denture-usage rate after insurance expansion, there has not been strong evidence of improvement in chewing ability.^15^^,^^16^ However, these findings were made in the immediate aftermath of the policy change, and any insurance effects on oral health outcomes in older adults need to be considered in the long-term.

Thus, we sought to adopt interrupted time-series analysis (ITSA) models to consider population changes in chewing difficulty over time. ITSA uses multiple consecutive pre- and post-intervention observations in a single population and incorporates time values.

Given these assumptions, we aimed to explore the causal inference of expanded dental insurance on older adults’ chewing ability using a multi-group ITSA model.

## METHODS

### Study design and main variable

We applied a multi-group ITSA, quasi-experimental study design to analyze the Korea National Health and Nutrition Examination Survey (KNHANES) data from 2007 to 2016–2018. The KNHANES is a nationally representative, cross-sectional survey conducted by the Korea Centers for Disease Control and Prevention (KCDC). We performed pre-intervention (2007 to 2011) and post-intervention (2012 to 2016–2018) comparisons.

The 2016–2018 data were collected at a single point that was provided by the KCDC, which could not identify each year’s data because of the small number of sample participants. For instance, in the 2011 data, 192 primary sampling units (PSUs) were drawn from approximately 200,000 geographically defined PSUs to consider urban and rural areas for the whole country, which comprised an average of 60 households, and of these, 20 final target households were sampled for each PSU using systematic sampling that includes approximately 10,000 individuals aged 1 year and over for each survey year.^17^

The study population comprised treatment and control groups. The treatment group was aged 65 years or older (aged 65 to 80 years); that is, those eligible for the elderly dental insurance benefit. The control group was aged younger than 65 years (aged 50 to 64 years); that is, those ineligible for the elderly dental insurance benefit. We restricted the sample to adults aged between 50 and 80 years to compare the same range of ages (65 ± 15 years) in both groups. Approximately 3,000 individuals were included for each year (Table 1). Approximately, of the participants, less than 3% were excluded because of missing values for our variables of interest (ie, chewing difficulty, age, sex, and study year). However, we confirmed that the missing values would not influence the results through reliability tests.

**Table 1.** Distribution of the study group by gender and year

Study year	Overall					Men					Women				
		
	*n* ^a^	<65^c^		≥65^d^		*n*	<65		≥65		*n*	<65		≥65	
		
		%^b^	95% CI	%	95% CI		%	95% CI	%	95% CI		%	95% CI	%	95% CI
2007	1,471	61	[0.58,0.65]	39	[0.35,0.42]	615	66	[0.61,0.71]	34	[0.29,0.39]	856	57	[0.53,0.62]	43	[0.38,0.47]
2008	3,188	62	[0.59,0.64]	38	[0.36,0.41]	1,305	66	[0.63,0.69]	34	[0.31,0.37]	1,883	58	[0.54,0.61]	42	[0.39,0.46]
2009	3,544	62	[0.60,0.64]	38	[0.36,0.40]	1,528	67	[0.64,0.69]	33	[0.31,0.36]	2,016	58	[0.55,0.61]	42	[0.39,0.45]
2010	3,131	62	[0.60,0.65]	38	[0.35,0.40]	1,364	67	[0.64,0.70]	33	[0.30,0.36]	1,767	59	[0.56,0.61]	41	[0.39,0.44]
2011	3,311	62	[0.60,0.65]	38	[0.35,0.40]	1,425	67	[0.64,0.69]	33	[0.31,0.36]	1,886	59	[0.55,0.62]	41	[0.38,0.45]
2012	3,292	62	[0.60,0.65]	38	[0.35,0.40]	1,390	67	[0.63,0.70]	33	[0.30,0.37]	1,902	59	[0.56,0.62]	41	[0.38,0.44]
2013	2,903	62	[0.60,0.65]	38	[0.35,0.40]	1,224	66	[0.63,0.70]	34	[0.30,0.37]	1,679	59	[0.56,0.62]	41	[0.38,0.44]
2014	3,093	62	[0.59,0.65]	38	[0.35,0.41]	1,301	66	[0.63,0.69]	34	[0.31,0.37]	1,792	59	[0.55,0.62]	41	[0.38,0.45]
2015	3,183	62	[0.60,0.65]	38	[0.35,0.40]	1,387	66	[0.63,0.69]	34	[0.31,0.37]	1,796	59	[0.56,0.62]	41	[0.38,0.44]
2016–2018^e^	7,116	53	[0.51,0.55]	47	[0.45,0.49]	3,060	52	[0.50,0.55]	48	[0.45,0.50]	4,056	54	[0.52,0.56]	46	[0.44,0.48]

CI, confidence interval.

^a & b^Unweighted number of subjects and weighted proportion (%) using svyset psu, strata(kstrata).

^c^<65 (aged under 65 years) denotes the control (ineligible) group aged 50 to 64 years.

^d^≥65 (aged 65 years and older) denotes the eligible (treatment) group aged 65 to 80 years.

^e^It considered a single point estimation.

The main outcome, chewing difficulty, was assessed using self-responses to the following in-person questions: “Do you have difficulty or discomfort when chewing food because of oral problems, involving teeth, dentures, or gums?” and “If you use dentures, please describe your experience with wearing them.” These questions rated “chewing difficulty” according to five options; severe difficulty, difficulty, some difficulty, little difficulty, and no difficulty. We used a cutoff to dichotomize the first two options (severe difficulty and difficulty) as chewing difficulty. Self-reported chewing ability is a valid and reliable measurement to evaluate mastication function for clinical tests and population studies among adults and older adults.^18^^–^^20^ The age-standard chewing difficulty rate was calculated to compare rates between time periods and to consider differences in the age structure of the two groups. The age-standardized population was included to convert the outcome into a rate and adjust for any potential changes in the population over time. The standard population from 2007 to 2016 was provided by the Korea National Statistical Office’s resident population registry.

### Statistical analysis

We performed a multi-group ITSA. Stratified analyses were performed to establish whether the policy effect on chewing difficulty varied by gender.

Ordinary least-squares (OLS) regression models designed to adjust for autocorrelation were employed for the multi-group ITSA, which included the treatment and control groups, testing our hypothesis that confounding omitted variables affect both groups similarly. We employed the “*newey*” Stata command, which estimates the coefficients to handle autocorrelation and possible heteroskedasticity. We implemented post-estimation time-series analysis to verify the post-intervention trend using the “itsa” Stata command specifically designed for time-series data.^21^

The multi-group analysis is expressed as follows:

$$\begin{array}{rl} Yt & =\beta 0+\beta 1Tt+\beta 2Xt+\beta 3XtTt+\beta 4Z\\ & \;+\beta 5ZTt+\beta 6ZXt+\beta 7ZXtTt+t \end{array}$$

**Figure 1.**
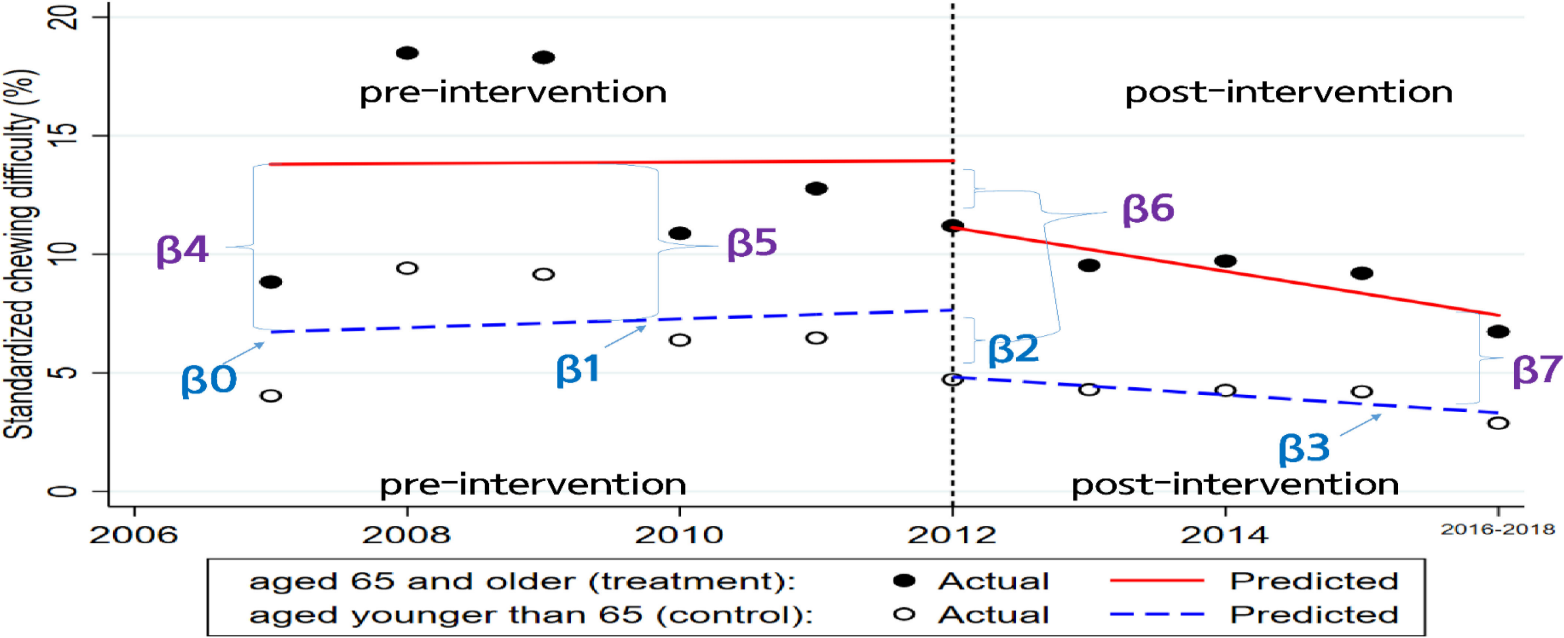
Visual depiction of a multi-group interrupted time-series design on chewing difficulty. Legend: β0 to β3 represent the control group (ineligible; aged younger than 65 years); β4 to β7 represent the treatment group (eligible; aged 65 years and older). β0: intercept; β1: slope prior to policy; β2: change in level in the period immediately following the policy initiation (compared with counterfactual); β3: difference between pre-policy and post-policy intervention slopes; β4: difference in the level between treatment and control prior to intervention; β5: difference in the slope between treatment and control groups prior to intervention; β6: difference in the level between treatment and control groups in the period immediately following intervention initiation; β7: difference between treatment and control groups in the slope after initiation of the intervention compared with pre-intervention.

Here, *Yt* is the chewing difficulty rate measured for each year *t*, *Tt* is the number of years since the start of the study, *Xt* is a dummy variable representing the intervention (pre-intervention: 0, otherwise 1), *Z* is a dummy variable that denotes group assignment (1 = treatment), and *XtTt*, *ZTt*, *ZXt*, and *ZXtTt* are all interaction terms of the previously described variables. These terms are illustrated in Figure 1.

The coefficients of the lower blue dashed line, β0 to β3, represent the control group, and the coefficients of the upper red bold line, β4 to β7, represent the treatment group. More specifically, β4 represents the difference in the level (intercept) of chewing difficulty between the treatment and control groups prior to the intervention, β5 represents the difference in the chewing difficulty slope (trend) between the treatment and control prior to the intervention, β6 indicates the difference in the chewing difficulty level between the treatment and control immediately following the intervention introduction, and β7 represents the difference in the chewing difficulty slope (trend) between the treatment and control after the policy initiation compared with the pre-intervention slope.^21^

We conducted sensitivity tests to verify the ITSA results’ robustness. These included an alternative control group (aged 20–35 years). We tested for autocorrelation to ensure model fit that accounts for the correct autocorrelation structure in the error distribution with a Cumby-Huizinga general test using the “actest” Stata command.^21^

We used Stata statistical software (Release 15; Stata Corp, College Station, TX, USA) for all statistical analyses.

### Ethics approval and consent to participate

This study used open-access data from the KNHANES conducted by the KCDC. All KNHANES respondents provided informed consent before participating. The KNHANES was approved by the KCDC Institutional Review Board (IRB). This is a publicly available secondary dataset. Our institute determined that the use of the KNHANES dataset does not meet the criteria for human subject research and is therefore exempt from IRB approval. It confirmed that all methods meet the relevant guidelines and regulations.

## RESULTS

The trend in chewing difficulty slopes downward over time in both those aged 65 years or older and those younger than 65 years after 2012. Figure 1 illustrates the interpretation of the related estimates provided in Table 2. Although there is an annual decrease of 0.93% in chewing difficulty for the treatment group (95% confidence interval [CI], −1.30 to −0.55) after the intervention, we found no statistical evidence for the insurance effect in β7 (−0.39%; 95% CI, −3.48 to 2.70).

**Table 2.** Lincom estimates for the multi-group design

Measure of interest	Model parameter	Overall		Men		Women	
		
		Point estimate	95% CI	Point estimate	95% CI	Point estimate	95% CI
**Between-group comparison^a^**							
Pre-intervention trend: control	β1	0.18	−1.24 to 1.61	0.35	−1.02 to 1.72	0.03	−1.46 to 1.52
Pre-intervention trend: treatment	β5 + β1	0.03	−2.66 to 2.72	0.20	−1.18 to 1.58	−0.28	−4.91 to 4.35

Difference pre-intervention: ​ treatment versus control	β5	−0.16	−3.20 to 2.89	−0.15	−2.09 to 1.80	−0.30	−5.17 to 4.56
Difference immediately following the intervention: ​ treatment versus control	β6	0.01	−8.61 to 8.63	0.61	−4.93 to 6.15	−0.78	−14.56 to 13.01
Post-intervention trend: control	β1 + β3	−0.38	−0.59 to −0.16	−0.34	−0.55 to −0.13	−0.41	−0.64 to −0.19
Post-intervention trend: treatment	β1 + β3 + β5 + β7	−0.93	−1.30 to −0.55	−0.42	−0.77 to −0.07	−1.69	−2.11 to −1.27
Difference post-intervention: ​ treatment versus control	β5 + β7	−0.55	−0.98 to −0.11	−0.08	−0.49 to 0.33	−1.28	−1.75 to −0.80
Difference pre- versus post-intervention: control	β3	−0.56	−2.03 to 0.91	−0.69	−2.10 to 0.72	−0.44	−1.97 to 1.09
Difference pre- versus post-intervention: treatment	β3 + β7	−0.95	−3.67 to 1.76	−0.62	−2.06 to 0.82	−1.41	−6.04 to 3.22
Difference pre- versus post-intervention: ​ treatment versus control	β7	−0.39	−3.48 to 2.70	0.07	−1.96 to 2.08	−0.97	−5.85 to 3.91

CI, confidence interval.

^a^Aged 65 years and older (treatment) vs aged younger than 65 years (control).

^b^Before 2012 (pre-intervention) vs after 2012 (post-intervention).

**Figure 2.**
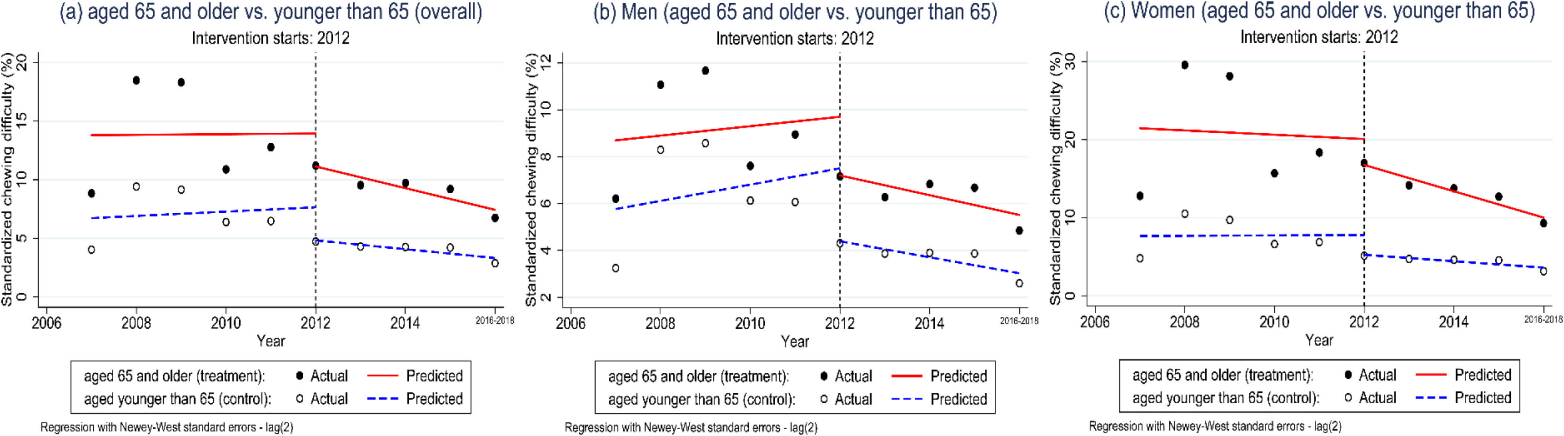
Interrupted time-series of chewing difficulty for the multi-group comparison design. Solid line (red): predicted standardized chewing difficulty trend of the treatment group (aged 65 years and older), Dashed line (blue): predicted standardized chewing difficulty trend of the control group (aged younger than 65 years).

Table 2 and Figure 2 show an increasing pre-intervention trend in annual chewing difficulty for both treatment and control groups. However, there is no difference in the pre-intervention chewing difficulty trend between the two, thus, similar pre-intervention levels and trends were identified. We confirmed that those aged under 65 years had an annual increase of 0.18% in chewing difficulty in β1 (95% CI, −1.24 to 1.61) between 2007 and 2012, and there was an annual increase of 0.03% in those older than 65 years (95% CI, −2.66 to 2.72) over the same period. The difference in trends for the pre-intervention period is thus −0.16% (95% CI, −3.20 to 2.89). In addition, there was no difference in the chewing difficulty level between the two groups immediately following the policy introduction in β6 (0.01%; 95% CI, −8.61 to 8.63); thus, similar levels immediately after the policy implementation were confirmed. The post-intervention trend was detected in both groups. Those older than 65 years had an annual decrease of 0.93% in chewing difficulty (in β1 + β3 + β5 + β7; 95% CI, −1.30 to −0.55) and those younger than 65 years had a decrease of 0.38% (95% CI, −0.59 to −0.16). The difference in the trends for the post-intervention period is −0.55% (95% CI, −3.20 to 2.89).

Stratifying the findings by gender shows some different features in the post-trend estimation; β1 + β3 + β5 + β7 and β5 + β7. We found that chewing difficulty decreases significantly in both genders annually after the intervention; women (−1.69%; 95% CI, −2.11 to −1.27) and men (−0.42%; 95% CI, −0.77, −0.07). However, compared with the control group (β5 + β7), these differences decrease more in women (−1.28%; 95% CI, −1.75 to −0.80) but not in men (−0.08%; 95% CI, −0.49 to 0.33) (Figure 2B and Figure 2C).

**Figure 3.**
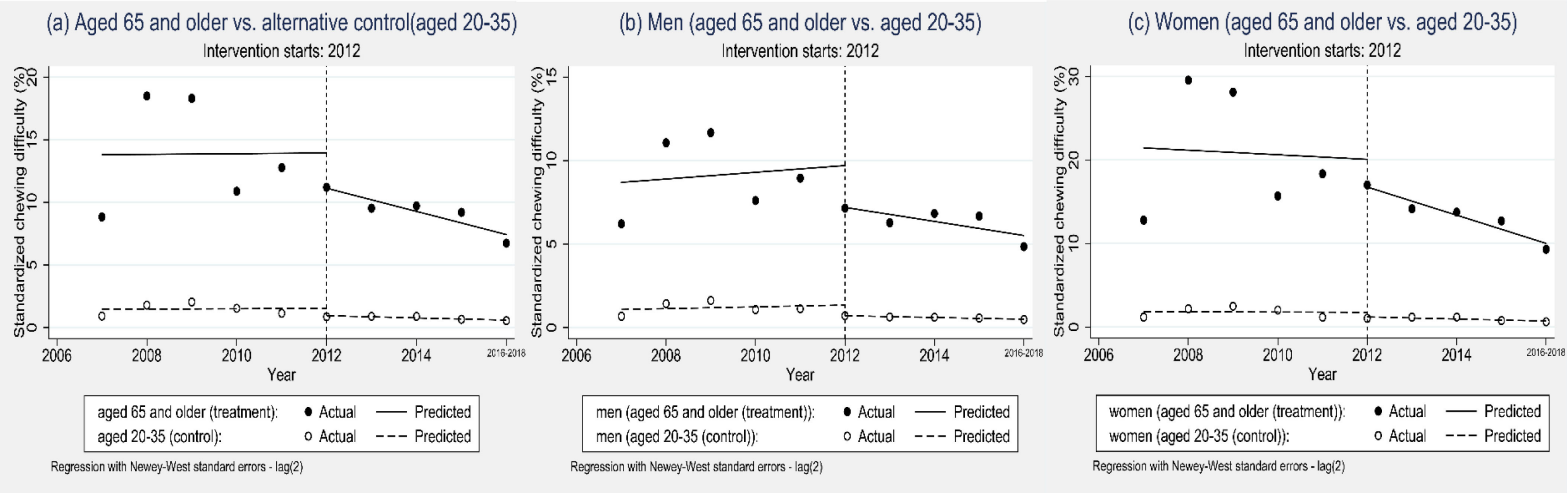
Sensitivity test for multi-group design using an alternative control group (aged 20 to 35 years). Solid line: predicted standardized chewing difficulty trend of the treatment group (aged 65 years and older), Dashed line: predicted standardized chewing difficulty trend of the alternative control group (aged 20 to 35 years).

We validated our multi-group ITSA model and confirmed the model fit because it showed a significance in lag (2) of the ITSA models (eTable 1 and eTable 2). Thus, we used the lag (2) model in the multi-group ITSA to adjust for autocorrelation. We validated our multi-group model with an alternative control group (aged 20 to 35 years) that showed results similar to our main results; there were no insurance effects on chewing difficulty in either gender (Figure 3).

## DISCUSSION

The major findings can be summarized as follows; first, the trend in chewing ability improved for older adults after the dental insurance policy. Second, however, we could not conclude that this improvement was due to the expansion in dental insurance because of a parallel improvement of chewing ability in both the eligible and ineligible groups. Finally, we found that chewing ability tended to improve in both older and younger adults regardless of the expansion in dental insurance coverage for older adults.

Different modeling techniques can provide different treatment effects.^22^ We employed OLS regression to ensure the robustness of our estimates with adjusting for autocorrelation.^21^^,^^23^ The slope change prior to the intervention indicates that an exogenous factor was already influencing the chewing difficulty rates and implies that the actual intervention (expansion of dental insurance) may simply be a continuation of those trends.^24^

We confirmed that our control group met the comparability criteria. Tests of the parameters describing the pre-intervention difference in intercepts (β4) and slopes (β5) comparing the treatment and control groups suggest that the groups were not significantly different with respect to pre-intervention levels of chewing difficulty and trends (*P*-values > 0.1 for both). The two parameters play a vital role in establishing whether the treatment group and controls are balanced on both the level and trend of the chewing difficulty in the pre-intervention period. We would expect similar levels and trends prior to the intervention if the difference in the mean baseline slope (β5) is significant and if that level remains the same throughout the duration of the observation period (Figure 2). Given this differential pattern of change in the baseline, one could argue that the control group was not comparable with the treatment group. In addition, the treatment effect for β7 might be biased.^25^ However, as shown in the regression results in Table 2 and illustrated in Figure 2, the treatment group is comparable with the controls in both baseline levels and trends. Thus, we confirmed that our model is valid for the comparison.

In the present analysis, we tested whether the expanded dental insurance benefits improved chewing ability in the eligible population. Although we found that chewing ability improved after the policy, a parallel trend was found in the ineligible control group. Hence, we conclude that exogenous factors were responsible for the improvements in chewing ability among older Korean adults.

Our findings did not highlight any gender differences in the treatment effect (β7) for chewing ability. However, previous studies found that women report more chewing difficulty than men among older adults.^26^^,^^27^ In addition, we found a generation gap among women in that there was a difference between women aged under 65 years and those aged 65 years and older (eFigure 1). Compared with the control group (aged under 65 years), women aged 65 years and older reported a significant annual decrease in chewing difficulty of 1.28%, whereas there was no significant decline for men after the policy expansion. However, there was no gender difference in the treatment effect because the control group for both men and women reported declining prevalence of chewing difficulty over the same period (Table 2 and Figure 2).

Enhanced chewing ability can be explained by improved oral health outcomes that relate to chewing functions (eg, number of teeth, periodontal health, and dental care access). Recent evidence supports this (eg, the increased number of present teeth, approximately three more teeth after 2012 than before).^28^^,^^29^ The severity of periodontal symptoms, including tooth mobility, gingival swelling, and gingival bleeding, decreased after 2012.^8^ Self-reported oral health also improved in the same period.^7^ These improved oral health outcomes were found in adults aged 65 years and older as well as those younger than 65 years.^7^^,^^8^^,^^28^^,^^29^ These trends suggest reasons for the improvement in chewing ability in both age groups and why it might not be attributable to the health policy change. This would seem to imply that, besides the changes in older adults’ dental insurance benefits, there have been other influences on improving oral health after 2012; for example, insurance extended coverage of dental scaling services for everyone over 19 years from 2013, which could affect the improvements in both groups.^6^^,^^11^^,^^30^ This could have led to improved dental care and oral health outcomes.

A recent study suggested that dental prosthesis may not always lead to positive outcomes such as chewing ability and oral health-related quality of life.^31^ Likewise there are influences beyond dental treatment that affect chewing ability. Important gaps remain in the evidence linking long-term effects of health policies to oral health. For example, further study is needed to probe whether trends in non-health care-related factors—such as food consumption practices or other social determinants—affect trends in oral health outcomes.

Some limitations in the ITSA model should be reported. Our results might have derived from exogenous factors beyond the expansion of dental insurance. Furthermore, we did not consider the actual uptake of dental insurance benefits. However, we did not present the results to confirm the assumption of compositional stability of the participants over time in this study, though we checked that the distributions of the participants remained stable on the factors linked to oral health outcomes.^7^^,^^16^ Self-reported outcomes might cause bias. However, such a bias would affect both groups during the study periods. Thus, we thought that the internal validity of our results would not be affected.

### Conclusion

Chewing difficulty decreased over time in both the eligible and ineligible groups after the expansion of dental insurance in South Korea. These trends appear to be attributable to forces external to changes in dental insurance benefits.

## APPENDIX A. SUPPLEMENTARY DATA

eFigure 1. Annual trends of the crude rate of chewing difficulty. Red line: the treatment group (aged 65 and older), Blue line: the control group (aged younger than 65). 2016 includes 2016 to 2018 year

eTable 1. Cumby-Huizinga test for autocorrelation

eTable 2. Sensitivity test for the lincom estimates in the multi-group design
