# Supplementary material for: Did Expanded Dental Insurance Improve Chewing Ability in the Older Korean Population? Results of an Interrupted Time-series Analysis
Source: J Epidemiol. 2022 May 5;32(5):215–20. doi: 10.2188/jea.JE20200417 (PMC8979917; doi:10.2188/jea.JE20200417)
Supplement: Supplementary file 1 [file je-32-215-s001.pdf]

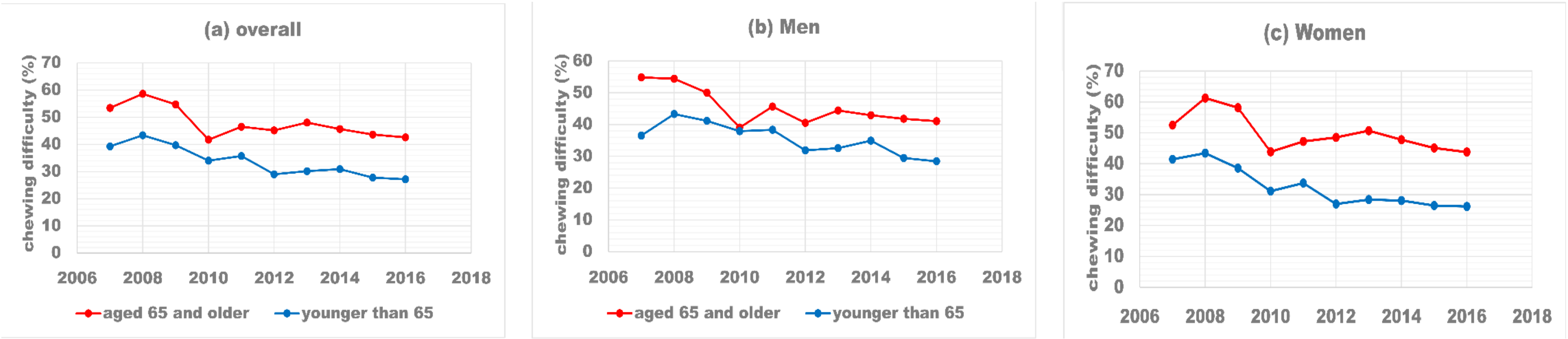

eFigure 1. Annual trends of the crude rate of chewing difficulty. Red line: the treatment group (aged 65 and older), Blue line: the control group (aged younger than 65). 2016 includes 2016 to 2018 year

eTable 1. Cumby-Huizinga test for autocorrelation

	lags	Chi^2	df	p-value	lag	Ch^2	dh	p-value
Overall								
	1-1	0.290	1	0.5904	1	0.290	1	0.5904
	1-2	6.810	2	0.0332	2	4.278	1	0.0386
	1-3	8.820	3	0.0318	3	0.147	1*	0.7014
	1-4	8.834	4	0.0654	4	0.001	1*	0.9760
Men								
	1-1	0.282	1	0.5955	1	0.282	1	0.5955
	1-2	7.687	2	0.0214	2	4.942	1	0.0262
	1-3	8.992	3	0.0294	3	0.191	1*	0.6622
	1-4	9.077	4	0.0592	4	0.036	1	0.8487
Women								
	1-1	0.308	1	0.5792	1	0.308	1	0.5792
	1-2	6.393	2	0.0409	2	3.917	1	0.0478
	1-3	8.801	3	0.0321	3	0.127	1*	0.7215
	1-4	8.802	4	0.0662	4	0.020	1*	0.8887

actest, lags(4)
* Eigenvalues adjusted to ensure a positive semidefinite matrix
H0: variable is MA process up to order q
HA: serial correlation present at specified lags >q
Test allows predetermined regressors/instruments
Test requires conditional homoskedasticity
H0: q=0 (serially uncorrelated) | H0: q=specified lag-1
HA: s.c. present at range specified | HA: s.c. present at lag specified

eTable 2. Sensitivity test for the lincom estimates in the multi-group design

Measure of interest	Model parameter	Overall		Men		Women	
		Point estimate	95% CI	Point estimate	95% CI	Point estimate	95% CI
**Between group comparison [a]**							
Pre-intervention trend: control	β1	0.02	-0.30 to 0.34	0.05	-0.18 to 0.28	-0.02	-0.45 to 0.41
Pre-intervention trend: treatment	β5 + β1	0.03	-2.66 to 2.72	0.20	-1.18 to 1.58	-0.28	-4.91 to 4.35
Difference pre-intervention: treatment versus control	β5	0.01	-2.69 to 2.72	0.15	-1.25 to 1.55	-0.26	-4.91 to 4.39
Difference immediately following the intervention: treatment versus control	β6	-2.22	-9.92 to 5.49	-1.86	-5.88 to 2.15	-2.77	-15.99 to 10.46
Post-intervention trend: control	β1 + β3	-0.08	-0.14 to -0.03	-0.05	-0.06 to -0.04	-0.12	-0.23 to -0.02
Post-intervention trend: treatment	β1 + β3 + β5 + β7	-0.93	-1.30 to -0.55	-0.42	-0.77 to -0.07	-1.69	-2.11 to -1.27
Difference post-intervention: treatment versus control	β5 + β7	-0.84	-1.22 to -0.46	-0.37	-0.72 to -0.02	-1.56	-1.99 to -1.13
Difference pre- versus post-intervention: control	β3	-0.10	-0.45 to 0.24	-0.10	-0.34 to 0.13	-0.10	-0.59 to 0.37
Difference pre- versus post-intervention: treatment	β3 + β7	-0.95	-3.67 to 1.76	-0.62	-2.06 to 0.82	-1.41	-6.04 to 3.22
Difference pre- versus post-intervention: treatment versus control	β7	-0.85	-3.59 to 1.89	-0.52	-1.98 to 0.94	-1.31	-5.96 to 3.35

CI, confidence interval.

[a] aged 65 and older (treatment) vs. aged 20–35 (control)

[b] before 2012 (pre-intervention) vs. after 2012 (post-intervention)
